# Case Report: Recurrent uterine inflammatory myofibroblastic tumor harboring *IGFBP5-ALK* fusion with sustained response to iruplinalkib

**DOI:** 10.3389/fonc.2026.1851744

**Published:** 2026-06-12

**Authors:** Ke Zhao, Min Hu, Runfeng Yang, Yi Huang

**Affiliations:** 1 Department of Gynecologic Oncology, Hubei Cancer Hospital, Tongji Medical College, Huazhong University of Science and Technology, Wuhan, Hubei, China; 2 Department of Pathology, Hubei Cancer Hospital, Tongji Medical College, Huazhong University of Science and Technology, Wuhan, Hubei, China

**Keywords:** *IGFBP5-ALK* fusion, inflammatory myofibroblastic tumor, iruplinalkib, tyrosine kinase inhibitor, uterine

## Abstract

Inflammatory myofibroblastic tumor (IMT) is an extremely rare mesenchymal tumor with approximately 50% of cases harbor anaplastic lymphoma kinase (ALK) gene rearrangements. The tyrosine kinase inhibitor (TKI) treatment has been increasingly common for IMTs with ALK fusions, but evidence for the efficacy of TKIs in uterine IMT is lacking. In this report, we present a case of patient with recurrent uterine IMT harboring *IGFBP5-ALK* fusion. The patient was initially misdiagnosed as uterine myxoid leiomyoma and then leiomyosarcoma after recurring. Then the patient was recommended ALK-TKIs treatment after detecting ALK rearrangement, and achieved complete response (CR) from a second-generation ALK-TKI inhibitor, iruplinalkib, after resistance to the first-generation inhibitor crizotinib. This is the first case of iruplinalkib achieved therapeutic success in uterine IMT, suggesting that a sequential ALK-TKIs with iruplinalkib could be an optimal targeted therapeutic strategy for ALK-rearranged IMTs.

## Introduction

Inflammatory myofibroblastic tumor (IMT) is a rare mesenchymal neoplasm which can manifest in any anatomical sites, but is more frequently located in the abdominal soft tissues and lung (1). IMT in the female genital tract is extremely rare, with the uterine being the most common localization (2, 3). Uterine IMT can occur at any age, but it typically presents in premenopausal women with abnormal uterine bleeding or dysmenorrhea (4). Despite most cases of uterine IMT are benign, a minority of cases with recurrence or metastasis have been reported in the literature, with the recurrence and metastasis rate of approximately 25% and 5%, respectively (5).

Diagnosis of uterine IMT can be challenging due to its morphological overlap with other more commonly seen mesenchymal tumors of the uterus, such as leiomyoma, leiomyosarcoma, and even endometrial stromal sarcoma (6). With the advancement of diagnostic tools such as immunohistochemical testing and molecular profiling, recognition of this tumor has improved. In fact, ALK immunohistochemistry, FISH and RNA sequencing have been proved to be highly sensitive and specific for the diagnosis of IMTs, with any positive result supports the diagnosis (7). In addition, tyrosine kinase inhibitor (TKI) treatment has been increasingly common for IMTs with ALK rearrangements. The first-generation ALK-TKI crizotinib and several second-generation ALK-TKIs, such as alectinib and ceritinib, have shown clinical efficacy in uterine IMT (8, 9). These results demonstrated that ALK-TKIs could be a potential therapeutic strategy for uterine IMT.

Herein, we present a case of recurrent uterine IMT harboring *IGFBP5-ALK* fusion who was treated with a sequential therapy of ALK inhibitors, and resulted in complete response from a second-generation ALK-TKI, iruplinalkib, after resistance to the first-generation inhibitor crizotinib. This is the initially reported case of uterine IMT achieved sustained response following treatment with iruplinalkib.

## Case description

A 58-year-old postmenopausal woman presented with progressive enlargement of the uterine myoma for two years and vaginal bleeding, and received a total hysterectomy and bilateral adnexectomy at an outside institution. The post-operative pathology of the mass suggested uterine myxoid leiomyoma by IHC staining: Vimentin (+), SMA (+), Desmin (+), Caldesmon (+), ER (partial+), PR (-), P16 (-), CD10 (-), CD34 (-), S-100 (-), STAT6 (-), P53 (partial+, wild type), AE1/AE3 (-), and Ki-67 (15% in hotspot area). The patient was followed up with routine postoperative clinic visits every 6 months and was diagnosed with recurrence by imaging one and a half years later. Then the patient underwent surgical resection at the same outside institution, and the postoperative pathology suggested uterine myxoid leiomyosarcoma.

**Figure 1:**
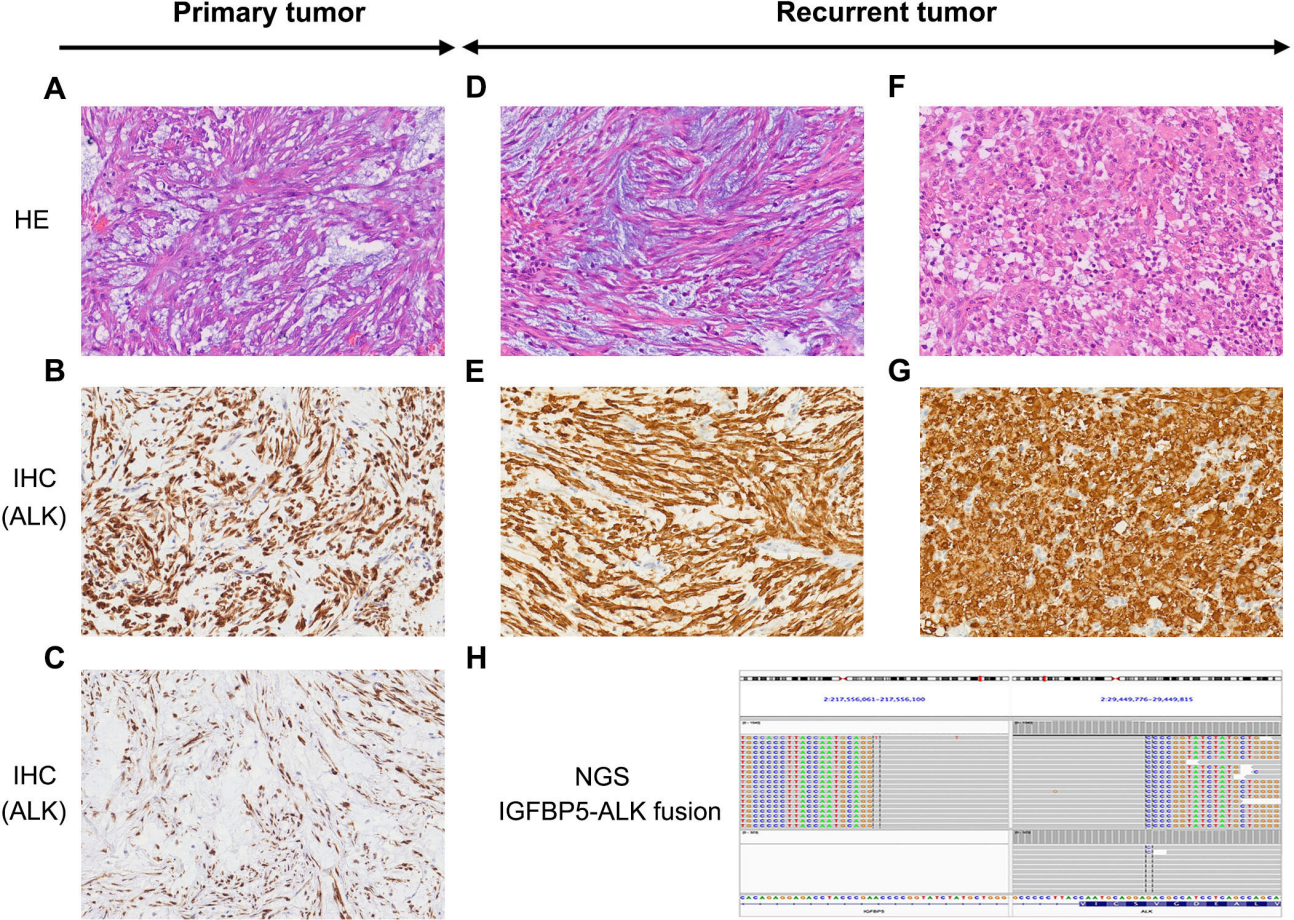
Pathological and molecular profiling. **(A)** Surgical resection specimen of the uterine mass showed diffuse spindle cells with abundant myxoid matrix **(B, C)** IHC staining confirmed the tumor cells were positive for ALK. Surgical specimen of the recurrent tumor showed spindle cells with abundant myxoid matrix and the positivity of ALK **(D, E)**, and epithelioid cells with positivity of ALK **(F, G)**. **(H)** NGS revealed *IGFBP5-ALK* fusion of the recurrent tumor. HE, hematoxylin and eosin; IHC, immunohistochemistry; NGS, next generation sequencing.

One month later, the patient came to our hospital for recommendations on therapy. We performed a pathology consultation on the external surgical resection specimens, and confirmed the histopathological diagnosis of uterine IMT with abundant myxoid matrix (**Figure 1A**), and IHC showed the tumor cells were diffusely positive for ALK (1A4) (**Figures 1B, C**). At the same time, a contrast-enhanced CT scan of the pelvis and abdomen at our hospital revealed multiple nodules in the mesentery, peritoneum, omentum, and retroperitoneal lymph nodes. Immediately afterwards, surgical resection of the pelvic and abdominal nodules was performed. Hematoxylin and eosin (HE) staining of the postoperative specimens showed two distinct areas: spindle and epithelioid. The spindle area is composed of spindle cells with abundant myxoid matrix (**Figure 1D**), while the epithelioid area is composed of sheets of epithelioid cells with vesicular nuclei and conspicuous nucleoli (**Figure 1F**). IHC showed both areas were strongly positive for ALK(D5F3) (**Figures 1E, G**). Ki-67 index in the spindle area was very low (about 1%), while in the epithelioid area it was higher (about 20%). Further molecular profiling via next-generation sequencing (NGS) (OncoScreenPlus, Burning Rock, Guangzhou, China; panel covering 520 human-related genes) was performed to detect the gene expression of the tumor sample, and an *IGFBP5-ALK* fusion was detected, the rearrangement breakpoint was between IGFBP5 exon 1 and ALK exon 19 (**Figure 1H**). Finally, the diagnosis was recurrent uterine IMT with *IGFBP5-ALK* gene fusion.

**Figure 2:**
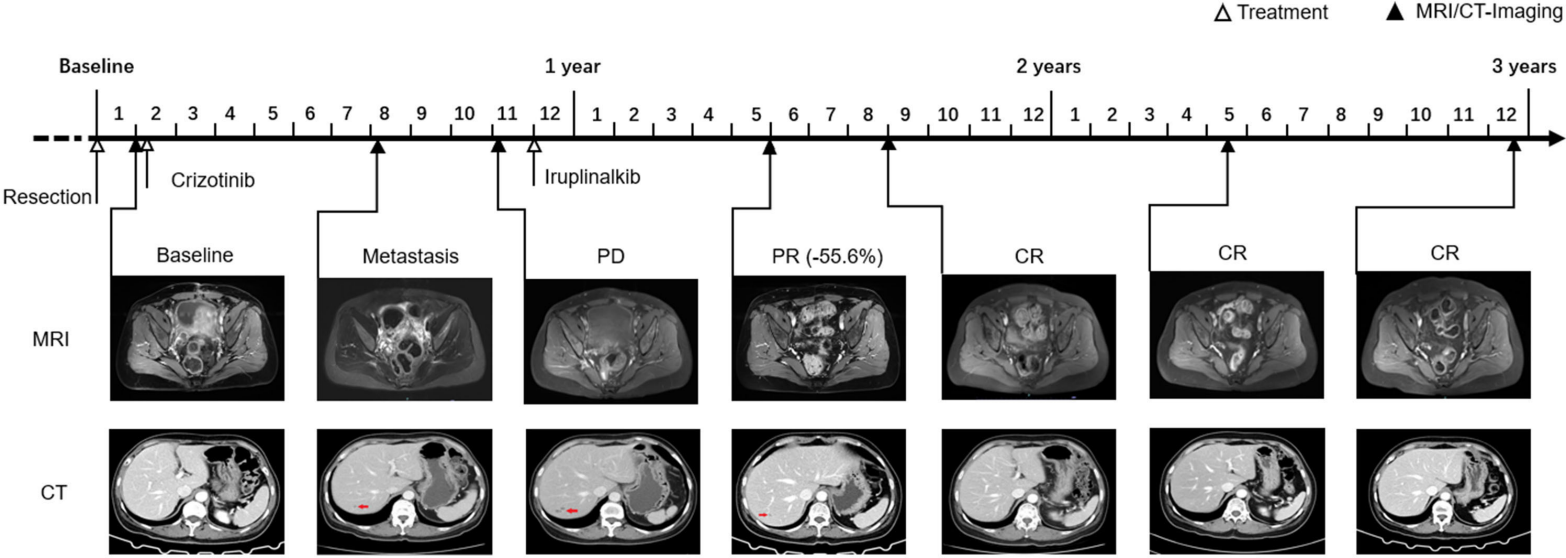
Overview of the treatment history of the patient, including treatment timeline, and images. PD, progressive disease; PR, partial response; CR, complete response.

Subsequently, the patient started crizotinib (250mg orally twice a day). Contrast-enhanced CT and pelvic MRI were performed to assess the efficacy of the therapy in the patient. Three months after the initial treatment, abdominal CT and MRI showed no residual or newly developed disease, but at the sixth month after treatment initiation, repeated CT showed a 2.8mm×2.2mm lesion in the liver indicating metastasis. The patient continued crizotinib treatment for three months, and re-examination of the abdominal CT revealed a marked enlargement in the size to 10.0 mm×4.8mm as well as another lesion measured 7mm×5.8mm developed in the liver. According to the New Response Evaluation Criteria in Solid Tumors (RECIST 1.1), the assessment for the radiographic changes were classified as progressive disease (PD) (10). After multidisciplinary team (MDT) discussion, the patient started a novel second-generation TKI at that time, iruplinalkib (60mg orally once a day). At the sixth month post-initiation, abdominal CT showed that the size of the liver metastatic lesion was markedly reduced and evaluated as partial response (PR). By 9 months after iruplinalkib initiation, the patient achieved complete response (CR), and maintained CR status for over 15 months. Imaging surveillance showed no evidence of progression of the disease through the time of this report (**Figure 2**). It’s worth noting that the common side effects of iruplinalkib such as hyperlipidemia, liver function abnormalities and skin rash are mild (Grade 1) and tolerable, the patient’s dosage administration of iruplinalkib did not adjust throughout the treatment process.

## Discussion

In this report, we presented a case of recurrent uterine IMT, which was initially misdiagnosed as a uterine myxoid leiomyoma and then as myxoid leiomyosarcoma after recurring. The patient was recommended treatment with ALK inhibitors after detection of the *IGFBP5-ALK* fusion by NGS. Consistent with observations of other researchers (8, 9), the patient achieved a long-term response to ALK-TKIs therapy. To the best of our knowledge, this is the first report of uterine IMT achieving a sustained response to the second-generation TKI, iruplinalkib, and it also illuminated several critical issues in the diagnosis and treatment of this tumor.

As far as we know, diagnosing uterine IMT is challenging due to its rarity and the significant morphologic and immunophenotypic overlap with other uterine tumors that exhibit myxoid changes (11). The spindle cell proliferation within myxoid stroma, accompanied by variable inflammatory infiltrates, including plasma cells, lymphocytes, and eosinophils, overlapped with myxoid smooth muscle neoplasms (myxoid leiomyoma and myxoid leiomyosarcoma) and endometrial stromal sarcoma. In addition, they express smooth muscle markers (e.g. SMA, desmin, caldesmon) and CD10, which can lead to the misdiagnosis of uterine IMT (12). Although ALK expression by IHC is highly sensitive and specific for the diagnosis of IMTs, it is not currently a routine screening test, since smooth muscle and endometrial stromal tumors are the most common uterine mesenchymal neoplasms. Kuisma et al. (13) revealed that about 0.3% of unselected leiomyomas were reclassified as IMTs based on positive ALK immunostaining. Parra-Herran et al. (14) reported four uterine IMTs that had been misclassified as leiomyosarcomas without detecting ALK initially among a series of 30 uterine myxoid leiomyosarcomas. Consistent with the above researches, the present case was initially misdiagnosed as myxoid leiomyoma because ALK IHC preliminary screening was not performed, reflecting overlook of this tumor.

*ALK* rearrangement by FISH is a useful and economical tool to confirm the diagnosis of IMT. In fact, approximately 50% of IMTs harbor an *ALK* gene rearrangement on chromosome 2p23 (6). However, cases of negative *ALK* rearrangement by FISH while positive for ALK by IHC have been reported (6, 15). These cases were shown by NGS to harbor *ALK* fusions, underscoring the importance of using multiple modalities, especially NGS for reaching the correct diagnosis. It has been identified by NGS in IMT that there were various fusion partners with *ALK*, e.g. *CLTC*, *RRBP1*, *RANBP2*, *IGFBP5*, *THBS1*, *TIMP3*, among which *IGFBP5* is the most prevalent fusion partner in uterine IMT (8). The *IGFBP5-ALK* fusion gene undergoes ligand-independent constitutive activation driven by the dimerization domain of IGFBP5, and it markedly activates downstream pathways, and promote excessive proliferation and apoptosis resistance of tumor cells (9, 15). In addition, Zhao et al. (9) demonstrated in their study that *IGFBP5-ALK* fusion was more frequently observed in recurrent uterine IMT and was easily misdiagnosed as leiomyosarcoma. The present study is consistent with the above observation.

ALK inhibitors are small-molecule tyrosine kinase inhibitors that competitively bind to the ATP-binding pocket of the ALK kinase domain, block ALK autophosphorylation and downstream oncogenic signaling, thereby suppressing tumor cell proliferation, invasion and inducing apoptosis (16). The application of ALK-TKIs to impede the *ALK*- mediated signaling pathways indispensable for the progression of IMT has dramatically altered the therapeutic paradigm for this disease. In 2010, Butrynski et al. reported the first case of patient treated with crizotinib achieved a sustained partial response, indicating that IMT with *ALK* fusions are responsive to ALK-TKI treatment (17). Subsequently, s series of clinical trials were carried out to investigate the efficacy of crizotinib in IMTs, and it was ultimately approved by the American Food and Drug Administration (FDA) for the treatment of IMTs with *ALK* fusions (18, 19). Alectinib is a second-generation TKI used after progression on or intolerance to crizotinib. Becht et al. (20) described an 18-year-old female with aggressive abdominal IMT who switched to alectinib since she was poorly tolerated on crizotinib. Still, the disease progressed several months later, and the patient achieved a long response to a third-generation TKI. The other second-generation TKIs, such as ceritinib and brigatinib, have also shown efficacy in sporadic case reports of ALK-positive IMT (21). These studies demonstrated the clinical benefits of sequential ALK-TKIs targeted therapy in IMT.

Iruplinalkib is a novel domestic second-generation TKI that is approved for the treatment of patients with locally advanced or metastatic ALK-positive non-small cell lung cancer (NSCLC) who have progressed after crizotinib (22). However, there was very limited data to date on its therapeutic potential for IMT. Recently, Zhang et al. reported a case of high-grade endometrial stromal sarcoma with *IGFBP5-ALK* fusion achieved satisfactory clinical outcome to iruplinalkib (23). The breakpoint of this rearrangement is between IGFBP5 exon 1 and ALK exon 19, suggests that the *IGFBP5-ALK* (I1; A19) fusion is likely iruplinalkib sensitive. Similarly, in our present study, NGS revealed the rearrangement breakpoint was also between *IGFBP5* exon 1 and *ALK* exon 19, and the patient achieved a complete response to iruplinalkib after developing resistance to crizotinib. This case report indicated the use of NGS can detect subtypes of driver genes, making the targeted therapy for different subtypes of driver gene more precisely.

To conclude, we present the first case of recurrent uterine IMT harboring *IGFBP5-ALK* fusion with a complete response from a second-generation ALK-TKI, iruplinalkib. Our report demonstrated the clinical benefits of sequential therapy with ALK inhibitors and an exceptionally long response to iruplinalkib in recurrent uterine IMT. Still, large-scale studies are needed to confirm the efficacy of iruplinalkib in IMT. Furthermore, this case indicated uterine IMT with *IGFBP5-ALK* fusion was prone to misdiagnosis, suggesting that the application of molecular testing, especially NGS in identifying the subtypes of driver genes is highly necessary for the correct diagnosis and targeted therapies of mesenchymal tumors.

## Data Availability

The original contributions presented in the study are included in the article/supplementary material. Further inquiries can be directed to the corresponding author.
